# Phytopathogenic *Rhodococcus* Have Diverse Plasmids With Few Conserved Virulence Functions

**DOI:** 10.3389/fmicb.2020.01022

**Published:** 2020-05-25

**Authors:** Elizabeth A. Savory, Alexandra J. Weisberg, Danielle M. Stevens, Allison L. Creason, Skylar L. Fuller, Emma M. Pearce, Jeff H. Chang

**Affiliations:** ^1^Department of Botany and Plant Pathology, Oregon State University, Corvallis, OR, United States; ^2^Center for Genome Research and Biocomputing, Oregon State University, Corvallis, OR, United States

**Keywords:** virulence plasmids, evolution, Gram-positive, *Rhodococcus*, cytokinins

## Abstract

*Rhodococcus* is a genus of Gram-positive bacteria with species that can cause growth deformations to a large number of plant species. This ability to cause disease is hypothesized to be dependent on a cluster of three gene loci on an almost 200 kb-sized linear plasmid. To reevaluate the roles of some of the genes in pathogenicity, we constructed and characterized deletion mutants of *fasR* and four *fas* genes. Findings confirmed that *fasR*, which encodes a putative transcriptional regulator, is necessary for pathogenesis. However, three of the *fas* genes, implicated in the metabolism of plant growth promoting cytokinins, are dispensable for the ability of the pathogen to cause disease. We also used long-read sequencing technology to generate high quality genome sequences for two phytopathogenic strains in which virulence genes are diverged in sequence and/or hypothesized to have recombined into the chromosome. Surprisingly, findings showed that the two strains carry extremely diverse virulence plasmids. Ortholog clustering identified only 12 genes present on all three virulence plasmids. *Rhodococcus* requires a small number of horizontally acquired traits to be pathogenic and the transmission of the corresponding genes, via recombination and conjugation, has the potential to rapidly diversify plasmids and bacterial populations.

## Introduction

Plasmids are major drivers in the evolution of bacterial pathogens of plants and animals. Acquired plasmids can innovate recipients by conferring novel functions such as the capacity to remodel host cells and suppress immunity (e.g., Reuter et al., 2014; The et al., 2016; Barton et al., 2018). Acquired plasmids can also coopt chromosomal genes and reprogram them to cause a change in lifestyle (Letek et al., 2010). Understanding plasmid ecology and evolution is critical but their study is challenging (Orlek et al., 2017). Plasmids are typically mobile, dispensable, and enriched in repeated sequences. As a consequence, plasmids are often variable in gene composition and their sequences can be difficult to identify in draft genome assemblies.

*Rhodococcus* is a genus of non-motile, non-sporulating, high G+C, mycolic acid containing Gram-positive bacteria (Larkin et al., 1998). Its members are cosmopolitan and can be found in terrestrial and aquatic environments, as pathogens of mammals, and as epiphytic and endophytic bacteria of plants. The genus is diverse, and, in a phylogeny, plant associated *Rhodococcus* strains are found in a clade separated from others by a long branch (Savory et al., 2017). Evidence suggests that plants may benefit from their symbioses with *Rhodococcus.* Strains have been associated to biocontrol and plant growth promoting activities (Francis et al., 2016; Hong et al., 2016; Savory et al., 2017). Moreover, *Rhodococcus* has been repeatedly identified in the microbiota of diverse species of plants, including Arabidopsis, rice, clover, and soybean (Delmotte et al., 2009; Bulgarelli et al., 2012; Knief et al., 2012; Lundberg et al., 2012; Vorholt, 2012; Bai et al., 2015). However, plant associated *Rhodococcus* bacteria can also be pathogenic. Strains carrying a virulence plasmid cause proliferations of buds or shoots, called leafy galls or witch’s brooms, to monocots, dicots, herbaceous, and some woody plants (Murai et al., 1980; Crespi et al., 1992; Putnam and Miller, 2007).

A virulence plasmid characterized from D188, a *Rhodococcus fascians* strain, is thought to be representative of those across the different species-level groups of plant pathogenic *Rhodococcus* (Savory et al., 2017). Plasmid pFiD188 is a linear replicon of approximately 200 kb (Francis et al., 2012). This plasmid has a cluster of genes that are either necessary for, or implicated in, virulence. The *fasR* and *fas* loci are necessary for virulence (Crespi et al., 1992; Temmerman et al., 2000). FasR is predicted to be a member of the AraC-type transcriptional regulator family. Despite its name, FasR does not appear to have a role in inducing the transcription of the *fas* operon (Temmerman et al., 2000). Of the *fas* operon, *fasA*, *fasD, fasE*, and *fasF* were concluded to be necessary for pathogenicity (Pertry et al., 2010). *In vitro* activity and functional annotations suggest three of these proteins are involved in the metabolism of cytokinins, which are best known as plant growth promoting hormones (Kieber and Schaller, 2014). FasD shows isopentenyltransferase (IPT) activity, one of the steps in cytokinin biosynthesis (Crespi et al., 1992). FasF exhibits phosphoribohydrolase activity and is a member of the Lonely Guy (LOG) family, a group of proteins that in plants catalyze the formation of free base forms of cytokinins (Pertry et al., 2010). FasE shows cytokinin dehydrogenase activity and can cleave and irreversibly inactivate cytokinins (Pertry et al., 2010). FasA is annotated as a putative cytochrome P450 monooxygenase. Also on pFiD188 and adjacent to *fasR* is the 10 gene *att* locus (Crespi et al., 1992; Maes et al., 2001). Mutants of *att* are reported to be attenuated in virulence and work suggested products of *att* are involved in signaling (Maes et al., 2001). Last, between *fasR* and *fas* are *mtr1* and *mtr2*, which encode methyltransferases (Francis et al., 2012; Radhika et al., 2015). Their predicted roles in virulence are described in a following section.

A population genomics study of phytopathogenic *Rhodococcus* suggested that there is low diversity among virulence plasmids (Savory et al., 2017). A total of 64 of 66 sequenced pathogenic strains have a plasmid that is similar in sequence and structure as pFiD188. Two exceptions are strains A21d2 and A25f, which because of the absence of homologs of most genes on pFiD188, were predicted to have virulence genes in their chromosomes (Creason et al., 2014). Additionally, the virulence genes of A21d2 are more diverged in sequence. The *fasR*_A21d2_ allele has a high number of single nucleotide polymorphisms and small insertion/deletion polymorphisms, relative to *fasR*_D188_. A21d2 also lacks homologs of *mtr1, mtr2*, as well as *fas* genes, and encodes a putative IPT-LOG hybrid protein (Creason et al., 2014). A25f, in contrast, has homologs of the cluster spanning from *att, fasR, mtr1, mtr2*, and *fas* on a single large contig. None of the other genes on the contig are homologous to those on pFiD188.

Even prior to the discovery of the *fas* genes, cytokinins were implicated in the virulence of *Rhodococcus* (Klämbt et al., 1966; Thimann and Sachs, 1966). One of the extant models predicts that a mixture of three cytokinin types, isopentenyladenine (iP), *trans*-zeatin (*t*Z), and *cis*-zeatin (*c*Z), as well as modified variants, are synthesized in a *fas*-dependent manner and secreted into plant cells to cause disease (Pertry et al., 2010; Stes et al., 2011). However, *t*Z is not reproducibly detected, *c*Z does not accumulate in a *fas*-dependent manner, and many of the cytokinin types accumulate in infected plants in patterns inconsistent with being causative (Eason et al., 1996; Galis et al., 2005; Creason et al., 2014; Jameson et al., 2019). A second model predicts that methyltransferases encoded by *mtr1* and *mtr2* function upstream of FasD to yield methylated cytokinins (Radhika et al., 2015). These novel cytokinins are predicted to be responsible for the ability of *Rhodococcus* to cause disease (Jameson et al., 2019). However, methylated cytokinins accumulate to low levels in bacteria and their levels do not vary in a plasmid-dependent manner (Pertry et al., 2009, 2010). A third model suggests that iP is the only type that is synthesized in a *fas* dependent manner but whether it directly causes disease has also been questioned (Creason et al., 2014; Jameson et al., 2019; Thapa et al., 2019).

Here, we used two approaches to generate resources to understand how *Rhodococcus* plasmids confer pathogenicity. We constructed and tested deletion mutants of *fasR* and four *fas* genes in strain D188. We also examined the role of *fasR* in A21d2. Results suggested that models of *Rhodococcus* virulence may have been informed by polar mutants of *fas*. We also generated higher quality genome sequences for pathogenic strains A21d2 and A25f. Resequencing uncovered two novel virulence plasmids in A21d2 and A25f that are distinct in structure and composition relative to pFiD188. Findings show that virulence of plant pathogenic *Rhodococcus* requires surprisingly few plasmid-encoded functions.

## Materials and Methods

### Bacterial Strains and Growth Conditions

Strains of *Rhodococcus* spp. were maintained on solid LB medium at 28°C or grown overnight in liquid LB medium with appropriate antibiotics at 28°C with shaking. *Escherichia coli* DH5α, 10-beta, and *ccdb-* were maintained on solid LB medium at 37°C or grown overnight in liquid LB medium at 37°C with shaking. Antibiotics were used at final concentrations of 25 μg/ml chloramphenicol, 25 μg/ml gentamicin, and 30 μg/ml kanamycin.

### Extraction of Nucleic Acids

To extract genomic DNA from *Rhodococcus*, the Wizard Genomic DNA Purification Kit was used, following the instructions of the manufacturer for Gram-positive bacteria (Promega Corporation, Madison, WI, United States). To extract plasmids from *E. coli*, the Qiagen QIAprep Miniprep Kit was used, following the instructions of the manufacturer (Qiagen Company, Hilden, Germany). To extract RNA from *Rhodococcus*, cells were pelleted and resuspended in 500 μl Tri-Reagent (Sigma-Aldrich) in 2 ml tubes containing Lysing Matrix B (MP Biomedicals, Irvine, CA, United States). A FastPrep24 (MP Biomedicals, Irvine, CA, United States) was used to lyse cells for 1 min at 6.0 M/s. After, 100 μl of chloroform was added to each tube. Tubes were vortexed briefly and centrifuged for 10 min at 4°C at maximum speed. The upper phase was transferred to a new tube and 350 μl of isopropanol was added. Samples were incubated at −20°C overnight and then centrifuged for 10 min at 4°C at maximum speed. The supernatant was decanted, samples were washed two times with 70% ethanol, and the RNA was resuspended in DEPC-treated H_2_O.

### DNA Cloning

Primers used for cloning are described in Supplementary Table S1. Over the course of the study, various methods, including Gateway (Invitrogen, Carlsbad, CA, United States), Gibson (NEB, Ipswich, MA, United States), and restriction enzyme digestion and ligation were used for cloning. Unless stated, DNA modifying enzymes were from NEB (Ipswich, MA, United States).

Gateway cloning was employed to make constructs used for generating deletion mutants. The pSelAct plasmid was linearized with *SmaI* and treated with CIP (van der Geize et al., 2008). The Gateway Cassette C.1 was released from pBluescript via an *Eco*RV digest. The two fragments were ligated by T4 DNA ligase to yield the plasmid, pSelAct-GW. Fragments corresponding to approximately 1.5 kb of the regions flanking the genes of interest were amplified in a two-step PCR. First, fragments were amplified using sequence-specific primers with partial *attB* sequences. Equimolar amounts of the two fragments were assembled in a second reaction and PCR with primers B1 and B2 were used to link the two fragments and complete the *attB* sequences. Assembled fragments were cloned via BP reaction into pDONR207 and cloned into pSelAct-GW using LR Clonase (Invitrogen, Carlsbad, CA, United States).

The *fasR* genes were cloned downstream of the L5 promoter by replacing either the tdTomato or GFP-encoding genes in vectors pJDC60 or pJDC165, respectively. For *fasR*_A21d2_ and *fasR*_D188_ (+ATG1), the coding sequences were amplified, products were digested with *Eco*RI and *Bam*HI and ligated to pJDC60 digested with the same restriction enzymes. Gibson cloning was used to generate the shorter variants of *fasR*_D188_ and the *fasR*_D188_ frameshift construct. The NEBuilder Assembly Tool v.1 (NEB, Ipswich, MA, United States) was used to design primers that amplified both pSelAct, the *fasR*_D188_ fragments, or the full length that also included a thymine to cause a frameshift. Fragments were amplified using KAPA HIFI, following the manufacturer’s instructions (Kapa Biosystems, St. Louis, MO, United States). Equimolar amounts of the two fragments were assembled using Gibson Assembly Mastermix and incubated for 30 min at 50°C.

PCR, restriction enzyme digestion, and Sanger sequencing were used to verify all constructs.

### Bacterial Transformation and Generation of Deletion Mutants

*Rhodococcus fascians* competent cells were prepared from overnight-grown 3 ml cultures. Cells were pelleted and washed twice with sterile, cold dH_2_O, followed by one wash with sterile, cold 10% glycerol. Cells were resuspended in 50 μl of 10% glycerol. Plasmid DNA (0.5–1 μg) was added to the cells, incubated on ice for 30 min and cells were electroporated in 1.0 mm gap cuvettes at 2.2 kV (Bio-Rad Micropulser, Hercules, CA, United States). Cells were resuspended in LB medium and incubated overnight at 28°C prior to plating on LB medium with appropriate antibiotics. For generating deletion mutants, merodiploids were selected for on LB plates with apramycin (50 μg/ml) and verified by PCR. Merodiploids were grown in liquid LB without antibiotics for 16–24 h at 28°C with shaking. Recombinants were selected for by growing cells for 5–10 days on mineral acetate medium with 5-fluorocytosine (100 μg/ml; van der Geize et al., 2008). PCR and Sanger sequencing of products were used to screen for and verify mutants.

### Quantitative Real-Time PCR

RNA was resuspended in a solution of 2 μl 10 × DNAse I buffer, 1 μl DNase I (Invitrogen, Carlsbad, CA, United States), 1 μl RNaseOUT (Invitrogen, Carlsbad, CA, United States) and 16 μl DEPC-treated H_2_O. Samples were incubated at room temperature for 15 min. DNase I was inactivated by adding 1 μl of 25 mM EDTA and heating samples for 10 min at 65°C. A total of 1 μl of DNAse-treated RNA from each sample was used directly in PCR to verify that there was no residual DNA sufficient for amplification. RNA was quantified using the Qubit RNA BR Assay Kit and a Qubit 4 Fluorimeter (Invitrogen, Carlsbad, CA, United States).

First-strand cDNA was synthesized from 1.0 μg total RNA, using qScript cDNA Supermix and following the manufacturer’s instructions (Quantabio, Beverly, MA, United States). cDNA equivalent to 25 ng of starting RNA template was used in 20 μl reaction volumes consisting of 10 μl Supermix, 0.5 μl each forward and reverse primers, 1 μl cDNA and 8 μl sterile RNase-free H_2_O. Reaction conditions were 95°C for 3 min followed by 40 cycles of 95°C, 10 s, 55°C 10 s, 72°C for 1 min on a Bio-Rad CFX96 Real-Time PCR Detection System (Bio-Rad, Hercules, CA, United States). Bio-Rad CFX Meistro software was used to analyze the data.

### Plant Inoculation Assays

*Nicotiana benthamiana* seeds were sterilized in a 20% bleach solution with a drop of polyoxyethylene sorbitan monolaurate (Tween 20) and shaken for 20–30 min. Seeds were washed twice with distilled water and resuspended in 1 ml sterile dH_2_O. Sterilized seeds were plated on MS agar medium (half-strength Murashige and Skoog medium, 0.5 M MES) and maintained vertically, at room temperature and constant light, for 3 days prior to inoculation. Overnight cultures of *Rhodococcus* were prepared in LB medium with appropriate antibiotics. Cells were pelleted, washed with sterile dH_2_O, and their concentration measured using a spectrophotometer. Suspensions were adjusted to OD_600_ = 0.5 in 100 mM MgCl_2_ prior to inoculation. Each seedling was inoculated with ∼3–5 μl of bacterial suspension. For each experiment, a negative control of an equivalent volume of 100 mM MgCl_2_ was included. Plates were allowed to dry briefly before being returned to the growing conditions described previously. Images of plants were taken 7 days post inoculation (dpi). Roots were measured via ImageJ (Schneider et al., 2012). All experiments were repeated at least three times with 40–50 seedlings inoculated per treatment. Data were analyzed, using one-way ANOVA followed by Tukey’s multiple comparisons test, as described in Savory et al. (2017). GraphPad Prism v. 7 was used for analyses (GraphPad Software, La Jolla, CA, United States).

For leafy gall assays, *N. benthamiana* seeds were sterilized as described above, plated on MS agar medium, and grown at room temperature under a 10:14 h light:dark cycle for 4 weeks. Bacteria were prepared as described above and 10 μl of OD_600_ = 0.5 of bacteria were dropped onto meristems of plants that had been pinched with forceps. Plants were returned to the growth chamber and inspected up to 28 dpi when images were taken. Infections included a mock treatment in which 10 μl of 100 mM MgCl_2_ was dropped onto meristems that had been pinched with forceps. At least three replicates were performed with 12 individuals per experiment.

### DNA Sequencing

For Sanger sequencing, PCR products were treated with 2.5 units of Exonuclease I and 0.25 U of Shrimp Alkaline Phosphatase and incubated at 37°C for 40 min and 80°C for 20 min. Products were sequenced on an ABI3730 capillary sequencing machine at the Oregon State University Center for Genome Research and Biocomputing (CGRB).

Strains A21d2 and A25f were multiplexed and sequenced with 1D chemistry (LSK109) on an Oxford Nanpore MinION Mk1b, run using a MinIT coprocessor. Native Ligation kit (SQK-LSK109) libraries were prepared according to the recommended native barcoding protocol and sequenced in multiplex. Fast basecalling and demultiplexing was performed live using Guppy v. 3.2.6 (Oxford Nanopore, Oxford, United Kingdom). Demultiplexing was either performed using Guppy during sequencing for A21d2 or using qcat v. 1.1.0 with the options “–trim –kit ‘NBD104/NBD114”’ for A25f (Oxford Nanopore, Oxford, United Kingdom). Illumina short reads were trimmed for adapters and quality, using the BBTools program bbduk v. 35.82 with Illumina barcode sequences and the options “ktrim = r k = 23 mink = 9 hdist = 1 minlength = 100 tpe tbo” (Bushnell, 2020). Long reads were *de novo* assembled using Canu v. 1.8 with the options “overlapper = mhap utgReAlign = true corMinCoverage = 0 useGrid = false corThreads = 4 maxThreads = 4 genomeSize = (Illumina-only assembly size) “–nanopore-raw” for A21d2 and with Flye v. 2.6 with the options “” for A25f (Koren et al., 2017; Kolmogorov et al., 2019). Unicycler v. 0.4.7 with options “–mode bold” and “–existing_long_read_assembly” was used to combine long-read assemblies with quality-trimmed Illumina reads to generate hybrid assemblies (Creason et al., 2014; Wick et al., 2017). Bandage v. 0.8.1 was used to assess the assembly graphs for completeness (Wick et al., 2015). Final assemblies were annotated using Prokka v. 1.14.0 “–gram pos –addgenes –rfam” (Seemann, 2014).

### Genome Analyses

Get_homologues v. 3.2.3 with MCL clustering was used to cluster orthologous genes between strains D188, A21d2, and A25f (Contreras-Moreira and Vinuesa, 2013). Each virulence plasmid gene was linked to a particular ortholog group (orthogroup) and used to identify homologs of genes on virulence plasmids.

The translated sequences of *attB, fasR*, and *fasD* were used in BLAST searches (default search parameters) against the NCBI nr database on February 10, 2020 to identify homologous sequences. The top 500 hits to full-length sequences for each were downloaded and aligned using MAFFT v. 7.402 e-ins-i (Katoh and Standley, 2013). Maximum likelihood phylogenies were constructed using IQ-TREE v. 1.6.12 with the options “–bb 1000 –alrt 1000” (Nguyen et al., 2015).

Genome sequences of *Rhodococcus* sp. Leaf225 and *Williamsia* sp. Leaf354 (accessions GCF_001426145.1 and GCF_001424365.1) were downloaded from NCBI. Sequences from pFiD188 and plasmids of A21d2 and A25f were used as queries in searches against these genome assemblies.

## Results

### Reevaluating the Role of *fas* Genes in Pathogenicity of *Rhodococcus*

The mechanistic basis by which *Rhodococcus* cause disease is unresolved, as findings from different research groups are incongruent (Stes et al., 2011; Creason et al., 2014; Jameson et al., 2019). In earlier attempts to repeat findings on the accumulation of cytokinins in culture grown *Rhodococcus*, we raised the possibility that the use of polar mutants may have contributed to inconsistencies between models (Creason et al., 2014). To address this, we constructed deletion mutants of *fasA, fasD, fasE*, and *fasF* in strain D188 to reevaluate the necessity of these genes in the pathogenicity of *Rhodococcus*.

**FIGURE 1:**
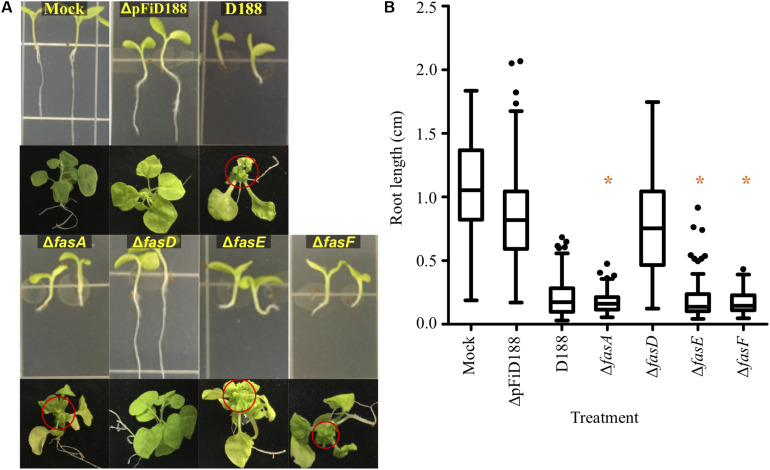
Most *fas* deletion mutants are pathogenic. **(A)** Wild type and deletion mutants of *Rhodococcus* strain D188 were inoculated onto seedlings or meristems of *N. benthamiana.* Red circles circumscribe leafy galls. This is a composite figure in which photographs were taken of plants treated in different experiments. Images of representative plants are shown. In all experiments, positive and negative control treatments were included. **(B)** At 7 dpi, roots were imaged and measured. Differences were compared relative to the corresponding D188 control group (* is not significant with a *p*-value threshold ≤0.05). Similar results were reproduced in at least two additional repeats of the experiments.

Phytopathogenic *Rhodococcus* causes symptoms to root systems and meristems of *N. benthamiana*. Seedlings infected with strain D188 were significantly stunted in root growth and terminally arrested at the cotyledon stage, relative to mock inoculated plants (Figures 1A,B). Meristems of plants directly inoculated with strain D188 had a proliferation of buds, typical of leafy gall disease (Figure 1A). Unexpectedly, *N. benthamiana* inoculated with Δ*fasA, ΔfasE*, or Δ*fasF* consistently exhibited symptoms similar to those on plants infected with pathogenic strain D188 (Figure 1). The roots of seedlings were stunted, and their lengths were not significantly different relative to the root lengths of plants infected with D188 (*p*-value ≤ 0.05). The three deletion mutants also reproducibly caused leafy galls on plants. Data suggest that *fasA, fasE*, and *fasF* are not individually necessary for *Rhodococcus* strain D188 to cause disease.

In contrast, plants inoculated with *ΔfasD* developed similarly to mock- and ΔpFiD188-inoculated plants (Figure 1). The latter treatment is with a non-pathogenic strain of D188 that lacks the virulence plasmid (Savory et al., 2017). Roots of *ΔfasD-*infected seedlings grew normally and were significantly longer than roots of D188-infected seedlings. Plants infected with *fasD* failed to show leafy gall disease symptoms when meristems were inoculated. However, quantitative reverse-transcriptase PCR (qRT-PCR) analyses showed that expression of the entire *fas* locus was compromised in the Δ*fasD* mutant (Supplementary Figure S1). The *fasA, fasE*, and *fasF* genes were expressed at significantly lower levels than in strain D188. It is also the case that the *fasD* coding sequence overlaps with *fasE* and in generating Δ*fasD*, we also compromised *fasE*. But as shown, the *ΔfasE* mutant is still pathogenic and dispensable for virulence (Figure 1). We also quantified expression of the *fas* genes in the original *fasD* insertion mutant relative to their expression in strain D188. Expression of *fasA, fasE*, and *fasF* is severely affected (Supplementary Figure S1). These data confirm that the *fas* operon is necessary for virulence, but we cannot unequivocally conclude that the *fasD* gene is necessary, as both an insertion within and deletion of, compromises expression of all examined *fas* genes at the locus.

### Refining the Annotation of *fasR*

**FIGURE 2:**
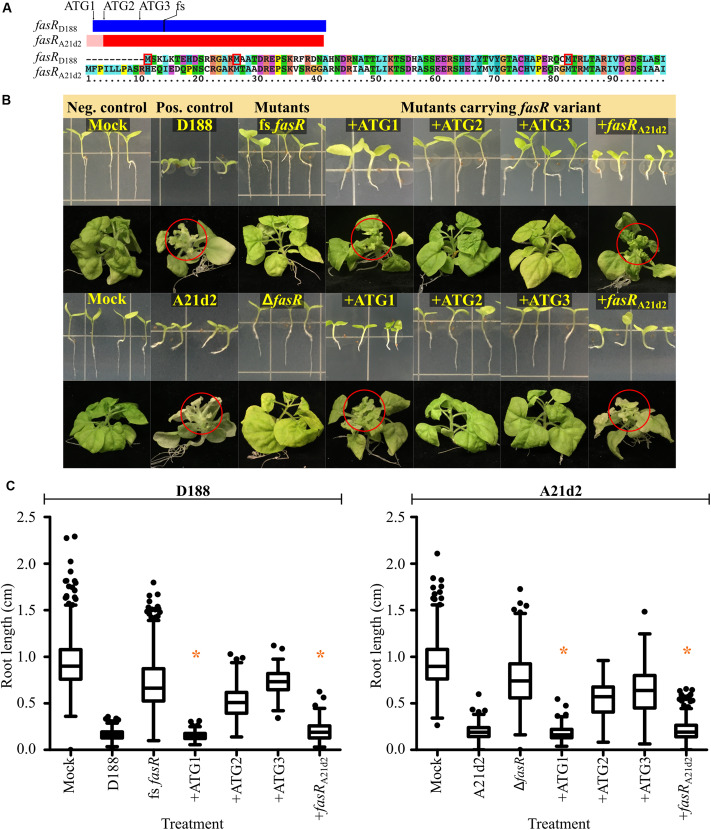
The *fasR* gene is necessary for virulence of D188 and A21d2. **(A)** Scaled schematic of the *fasR* coding sequences of D188 (blue) and A21d2 (red). The first three in-frame ATG codons of *fasR*_D188_ are shown. The location of the single base insertion introduced to cause a frameshift (fs) in *fasR*_D188_ is also indicated. A possible 5′ extension to *fasR*_A21d2_ is depicted by a pink-colored box. An alignment of the first ∼100 amino acids of the FasR variants of D188 and A21d2 is shown below the schematic. The first three methionine residues of FasR of D188 are boxed in red. **(B)** Wild type and mutant strains of D188 and A21d2 were inoculated onto seedlings or meristems of *N. benthamiana.* Red circles circumscribe leafy galls. Images of representative plants are shown. This is a composite figure in which photographs were taken of plants treated in different experiments. Images of representative plants are shown. In all experiments, positive and negative control treatments were included. **(C)** At 7 dpi, roots were imaged and measured. Differences were compared relative to the corresponding D188 or A21d2 control groups (* is not significant with a *p*-value threshold ≤0.05). Similar results were reproduced in at least two additional repeats of the experiments.

Comparisons of the sequences of the 64 pFiD188-like plasmids identified a sequencing mistake in the original annotation of *fasR* that was carried forward in analyses (Temmerman et al., 2000; Francis et al., 2012). A guanine base was inserted upstream of the annotated ATG of *fasR*_D188_. Correcting the error revealed two in frame ATG codons 213 (ATG1) and 168 (ATG2) nucleotides 5′ to the originally annotated start codon (ATG3; Figure 2A; Creason et al., 2014). Automated annotation programs identified ATG2 as the start because it has a strong ribosome binding sequence. Of the 12 nucleotides immediately upstream of ATG2, 11 are guanine or adenine bases. We cloned the three potential coding sequences, starting from each of the first three ATG codons, downstream of a mycobacteriophage L5 promoter that demonstrably constitutively expresses in *Rhodococcus*. We also generated a new mutant allele of *fasR*_D188_ (fs *fasR*_D188_) by introducing a nucleotide into the coding sequence to cause a frameshift mutation because previous studies used a deletion mutant that removes a portion of the neighboring *attH* gene (Figure 2A; Temmerman et al., 2000). On plants, fs *fasR*_D188_ failed to inhibit root development and failed to cause leafy gall disease, confirming the necessity of *fasR* for pathogenicity (Figures 2B,C).

We next tested each of the three possible *fasR*_D188_ coding sequences to determine which is sufficient to complement the mutant. The mutant strain carrying the longest coding sequence (+ATG1) inhibited root growth to the same degree as strain D188 (Figures 2B,C). In contrast, inoculations with strains carrying the *fasR* coding sequence starting from the second (+ATG2) or third ATG (+ATG3) codon resulted in root lengths that were intermediate to those of mock- and D188-inoculated plants. Importantly, on meristems of plants, the mutant strain carrying +ATG1 caused leafy gall symptoms whereas fs *fasR*_D188_ carrying either of the shorter variants caused no visible disease symptoms. Either ATG1 is the start of the coding sequence or sequences upstream of ATG2 or ATG3 are necessary for stable expression of *fasR.*

Of the 66 alleles of *fasR* that have been sequenced, the A21d2 allele is the most diverged, and automated programs predicted it to be 18 codons shorter relative to the D188 allele (Figure 2A; Creason et al., 2014). There is another in frame ATG 75 nucleotides upstream from the ATG originally annotated as the start codon (pink region in Figure 2A). This aligned to another ATG further 5′ but out of frame to ATG1-3 of *fasR*_D188_. We relied on the originally annotated start codon of *fasR*_A21d2_. We generated and tested a *fasR* deletion mutant in A21d2 and confirmed its necessity in pathogenicity (Figures 2B,C). The Δ*fasR* mutant of A21d2 could be complemented with *fasR*_A21d2_ expressed from the L5 promoter. We next tested whether *fasR*_A21d2_ and *fasR*_D188_ alleles could complement *fasR* mutants in either strain. Despite the diversity, *fasR*_A21d2_ could complement the *fasR* frameshift mutant of D188. Similarly, +ATG1 could complement Δ*fasR* of A21d2 but neither +ATG2 or +ATG3 could do so.

### Discovering Two Novel Virulence Plasmids in Phytopathogenic *Rhodococcus*

**FIGURE 3:**
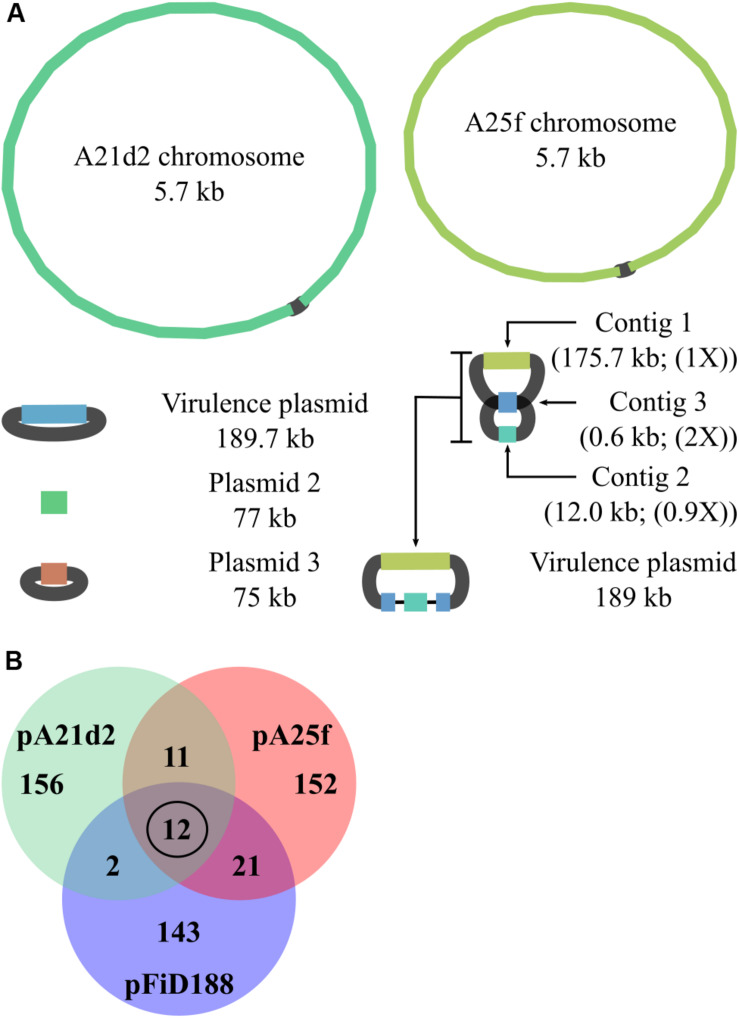
Pathogenic strains A21d2 and A25f have novel virulence plasmids. **(A)** Graphs of hybrid assembled genome sequences of A21d2 and A25f. Sizes of the replicons are shown. For the pA25f, a predicted structure is presented. The relative depth of coverage of plasmid contigs is shown in parentheses. **(B)** Venn diagram of gene homolog similarity. The sequences of all genes on the plasmids of A21d2, A25f, and D188 were translated and clustered on the basis of homology.

To gain more insights into the genomic context of virulence loci in strains A21d2 and A25f, we used Oxford Nanopore sequencing to generate long reads and combined them with the original Illumina-derived short reads. Nanopore sequencing produced 418,684 and 45,552 reads, respectively, with a mean read length of 7.8 and 4.0 kb. The longest reads for A21d2 and A25f were 112 and 106 kb, respectively. The A21d2 chromosome is 5,667,798 bp, and the A25f chromosome is 5,706,031 bp. Both strains have plasmids. Based on the assembly graph, two of the three plasmids in A21d2 are circular molecules (Figure 3A). The single plasmid in A25f is fragmented into three contigs (Figure 3A). Analysis suggested contig 3, which is 0.6 kb in length, has a repeated sequence that misassembled into one. The sequences are predicted to flank the other small contig and join to the largest plasmid contig to form a single circular molecule.

To our surprise, the virulence loci in each of these strains are located on circular plasmids. The virulence plasmid of A21d2, referred to as pA21d2, is 189,682 bp and has a cluster, from A21d2_05402 to A21d2_0514, of genes that are homologs of the 10 *att* genes, *fasR*, and IPT-LOG-encoding gene. The latter is annotated as *miaA.* MiaA is a tRNA-isopentenyltransferase (tRNA-IPT) that modifies base 37 on a subset of tRNAs (Schweizer et al., 2017). The annotations for tRNA-IPTs and IPTs are frequently misassigned by automated annotation programs (Takei et al., 2001; Lindner et al., 2014). Consistent with previous reports, there are no homologs of *fasA, fasB, fasC, fasE, mtr1*, or *mtr2* downstream of *fasR* or anywhere else on any of the replicons of A21d2. Instead, adjacent and downstream of *fasR* on pA21d2 is a gene that encodes a putative radical SAM enzyme (Creason et al., 2014). In pA25f, from A25f_05486 to A25f_05504, there are homologs of *att, fasR*, and *fasA-F* that are conserved in sequence and order to the corresponding region of pFiD188.

**FIGURE 4:**
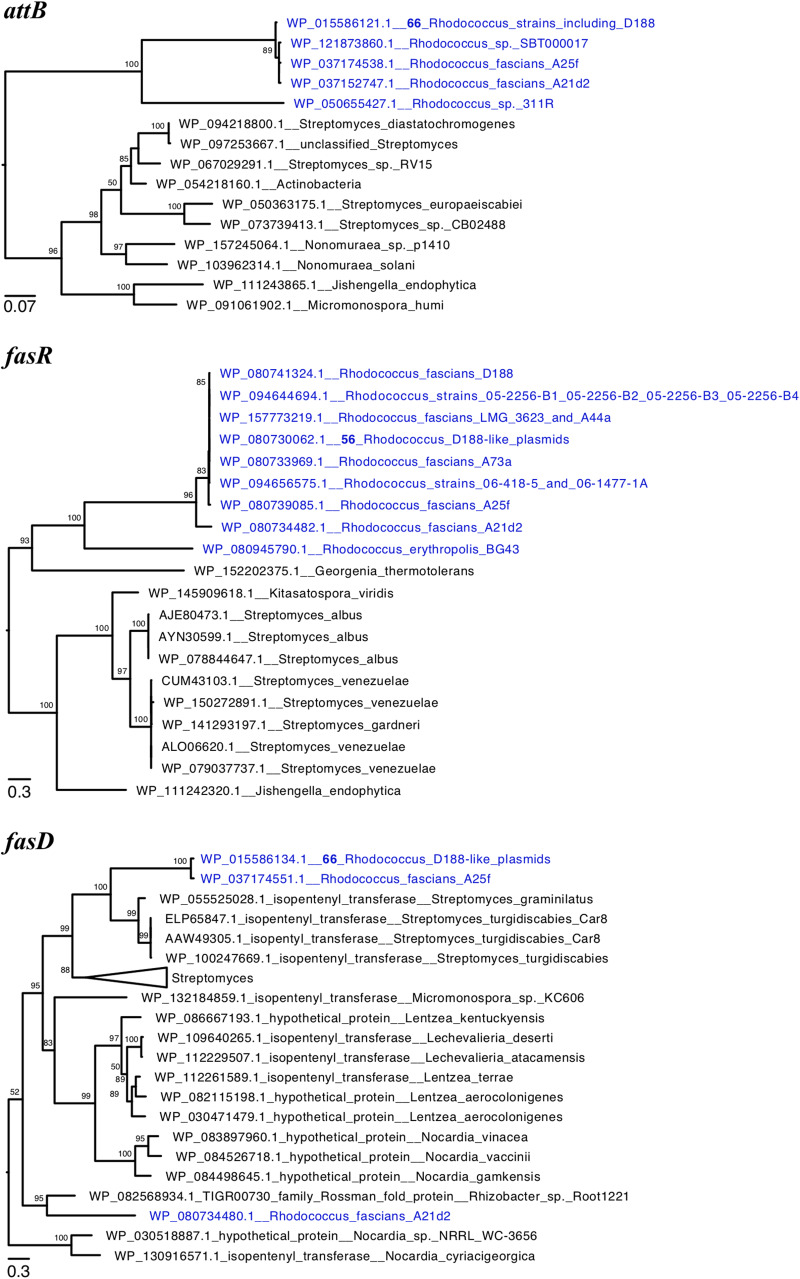
Phylogeny of virulence genes of plant pathogenic *Rhodococcus*. Maximum likelihood phylogenetic trees of translated sequences of **(A)** *attB*, **(B)** *fasR*, and **(C)** *fasD.* The trees were midpoint rooted and a subset of clades of *Rhodococcus* virulence proteins are shown. Tips with identical sequences are indicated. Large clades were collapsed and labeled. Bootstrap values greater than 50 are shown.

We constructed phylogenetic trees for the translated sequences of *attB, fasR*, and *fasD* (Figure 4). AttB of pA21d2 and A25f grouped with those of plant pathogenic *Rhodococcus*, suggesting *attB* and likely all *att* genes are derived from a recent common ancestor (Figure 4A). In the FasR and FasD trees, the sequences from A21d2 are more distant and their corresponding genes likely emerged from a more ancestral source or diverged via recombination (Figures 4B,C; Creason et al., 2014). In contrast, the pA25f variants of these two virulence protein sequences grouped with others, supporting a scenario in which a homologous fragment spanning from A25f_05486 to A25f_05504 (*att* through *fas*) is a recombinogenic unit that can be shared among diverse *Rhodococcus* plasmids.

Two publicly available genome sequences of non-plant associated *Rhodococcus* also have homologs of *attB* (Figure 4A). Strain 311R was isolated from hydrocarbon-contaminated soils and strain SBT000017 was isolated from marine organisms (Macintyre et al., 2014; Ehsani et al., 2015). In a species phylogeny, strain 311R clustered with *Rhodococcus erythropolis*, distant from the major clade with plant pathogenic strains (Savory et al., 2017). In contrast, strain SBT000017 clusters with plant pathogenic strains and is in the same clade as strain D188. Both 311R and SBT000017 have a complete *att* locus but no homologs of *fasR, mtr1, mtr2, fas*, or homologs that encode the putative radical SAM enzyme or IPT-LOG and are inferred to be non-pathogenic. In strain 311R, the translated *attB* sequence is divergent (71% amino acid identity) relative to the D188 sequence. Its *att* locus is adjacent to integrase-encoding genes and followed by genes of unknown function and located in the chromosome between *aspC_1* and *pur*. In strain D188, *aspC_1* and *pur* are immediately adjacent to one another, suggesting that strain 311R acquired a small island that circumscribes the *att* locus. In strain SBT000017, the translated *att* sequences are very similar to those of pFiD188 and range from 97.7 to 99.5% amino acid identity. The *att* locus is located on yet another novel plasmid.

The percentage of genes with homologs among pFiD188, pA21d2, and pA25f is low and suggest that they are unrelated plasmids that acquired the capacity to confer virulence to *Rhodococcus*. We translated the gene sequences of the three plasmids and clustered the sequences on the basis of homology. We identified only 12 gene sequences that are common to all three plasmids. Ten corresponded to the genes of the *att* locus and one is *fasR* (Figure 3B and Supplementary Table S2). The last encodes a predicted hypothetical protein (AMY56290.1). Other than these 12 genes, the number of other genes with homologs common to pairs of virulence plasmids is low, ranging from 2 to 20. Given that pFiD188 and A25f are identical in gene composition from *att* through *fas*, it was not surprising that there is slightly higher similarity between these two plasmids. No gene on pFiD188, implicated in plasmid maintenance or conjugation, had a homolog on the other two plasmids, indicating that pFiD188-like, pA21d2, and pA25f plasmids have separate origins (Francis et al., 2012).

### Mining Genome Sequences for Virulence Plasmids

Last, we reexamined the genome sequences of *Rhodococcus* sp. Leaf225 and *Williamsia* sp. Leaf354 for evidence of virulence plasmids (Bai et al., 2015). These two strains, despite one belonging to a different bacterial genus, were used to support the model that pathogenic *Rhodococcus* synthesizes novel methylated cytokinins and should therefore minimally have homologs of *mtr1, mtr2*, and *fasD* (Jameson et al., 2019). Previous work using *att, fasR*, or *fas* sequences from pFiD188 in BLAST queries failed to identify homologs in their genome sequences (Savory et al., 2017). To test the possibility that strains Leaf225 and Leaf354 have homologs of the novel plasmids uncovered in this study, we used all gene sequences from all three plasmids as queries in BLAST searches against the genome sequences of Leaf225 and Leaf354 (Supplementary Table S3). In addition to little evidence for virulence plasmids, we found no evidence that Leaf225 and Leaf354 have homologs of the genes core to all three plasmids, genes encoding Mtr1, Mtr2, the putative radical SAM enzyme or the IPT-LOG hybrid. When we lowered the thresholds in searches, we identified hits (<34–40% amino acid similarity) to Mtr1 and Mtr2 in Leaf225 (WP_027503960.1 and WP_056447883.1) and Leaf354 (WP_055787211.1, WP_055786245.1, WP_055790396.1, WP_055793823, WP_082501997.1, and WP_156378120.1 of Leaf354). However, use of these eight sequences as queries in reciprocal searches against the D188 genome sequence yielded better hits to genes in the chromosome, not pFiD188. Data suggested that Leaf225 and Leaf354 lack homologs of genes necessary for, or associated to, virulence.

## Discussion

Virulence plasmids are essential for *Rhodococcus* to cause leafy gall disease to plants (Crespi et al., 1992; Pertry et al., 2009, 2010; Stes et al., 2011; Creason et al., 2014; Savory et al., 2017). Using long-read sequencing technology, we discovered two new virulence plasmids in plant pathogenic *Rhodococcus* (Figure 3A). Comparisons showed that there are very few conserved genes among the three known plasmids and our genetic analysis of mutants suggested these plasmids require very few plasmid-encoded functions to confer virulence. However, it has been suggested that additional pFiD188-encoded genes are required for pathogenicity of strain D188. A cosmid that covers *att* and *fas* regions from pFiD188 is insufficient for pathogenicity and it has also been suggested that *fas* expression requires another plasmid-encoded transcription factor for full expression (Crespi et al., 1992; Temmerman et al., 2000). Our analyses suggest if other plasmid-encoded functions are required for pathogenicity, that these functions are conferred by genes highly diverged in sequence.

Clustering of a small number of genes necessary for pathogenicity is a low barrier for recombination to convert non-virulence plasmids to virulence plasmids. The discovery of a novel plasmid bearing an *att* locus in SBT000017 raises the potential that the *att* locus can mediate recombination among distinct plasmids. This is one plausible mechanism to explain how pA25f and pFiD188, plasmids that differ dramatically in composition, exchange virulence loci (Figure 4). Spread of virulence loci among plasmids and spread of virulence plasmids among strains, combine to accelerate the diversification of pathogenic *Rhodococcus* populations (Savory et al., 2017). This has significant implications on disease ecology because diversification can drive the emergence of disease on novel hosts, increased aggressiveness, and further increased spread (McDonald and Stukenbrock, 2016).

Of the functions inferred to be necessary for virulence, *fas-*derived cytokinins have long been central to models (Stes et al., 2011; Creason et al., 2014; Jameson et al., 2019). It has not been definitively demonstrated that *fasD* is necessary for pathogenicity. Nonetheless, the data are consistent with IPT being encoded on all three plasmids and collectively point to IPT activity as the *fas-*encoded function that is necessary for virulence (Figure 1). It remains unresolved as to whether the products of IPT are synthesized to be secreted to manipulate plants. One source of disagreement among virulence models is the use of polar mutants, which unknowingly impacted conclusions regarding the role of each *fas* gene in virulence and the synthesis of specific cytokinin types (Desomer et al., 1991; Crespi et al., 1992; Pertry et al., 2010). The original *fasD* insertion mutant has polar effects and there is also potential that other previously published *fas* mutants have polar effects (Supplementary Figure S1). The *fasA* mutant was identified from the same insertion library as the *fasD* mutant and was carried forward in subsequent studies. The *fasE* and *fasF* mutants were made via insertional mutagenesis (Pertry et al., 2010).

Another source of disagreement stems from the challenges with directly testing virulence models. During infection, it is difficult to show cytokinins originate from the microbe. Both partners in the symbiosis produce the hormone. Unless the pathogen is able to synthesize overwhelming quantities of cytokinins, the majority of detected cytokinins are likely derived from the host because the biomass of the plant vastly exceeds that of the pathogen. In culture, *Rhodococcus* cells produce picomolar to nanomolar amounts of cytokinins (Pertry et al., 2009, 2010; Creason et al., 2014). It is also the case that cytokinins are involved in plant immunity and levels may change because of responses of the plant, not directly because of the pathogen (Choi et al., 2010; Großkinsky et al., 2011; Jiang et al., 2013; Albrecht and Argueso, 2017). Further confounding analyses is that plants and bacteria have alternative sources of cytokinins. A subset of tRNAs in plants and bacteria are modified with cytokinins, hypothesized to promote more efficient translation (Schweizer et al., 2017). The degradation of tRNAs is a source of cytokinins that have yet to be accounted for to understand how *fas-*derived cytokinins contribute to virulence (Matsubara et al., 1968; Cherayil and Lipsett, 1977; Murai et al., 1980; Kasahara et al., 2004).

Findings here also cloud the role of methylated cytokinins in the virulence of *Rhodococcus*. In contrast to the conclusion that all pathogenic strains have *mtr1* and *mtr2*, we confirmed that A21d2 lacks *mtr* genes (Jameson et al., 2019). However, A21d2 encodes a member of the radical SAM enzyme family, and it is possible that this enzyme could contribute to the synthesis of methylated cytokinins (Zhang et al., 2012). Regardless, while levels of methylated cytokinins of plants change in response to infection, increases do not strictly correlate to infection by strains carrying homologs of genes demonstrably necessary for causing disease, or homologs of *mtr* genes implicated in the synthesis of methylated cytokinins (Supplementary Table S3). Strains Leaf225 and Leaf354 were originally isolated from asymptomatic Arabidopsis plants (Bai et al., 2015). Furthermore, *Rhodococcus* Leaf225 clusters in a clade with plant associated *Rhodococcus* strains in which no pathogenic isolates have been uncovered. *Williamsia* sp. Leaf354 is not a member of the *Rhodococcus* genus.

There are several examples that suggest microbe-synthesized cytokinins may have roles other than in virulence. *Claviceps purpurea* encodes an IPT-LOG hybrid protein and its corresponding mutant is unaffected in virulence and is altered in development (Hinsch et al., 2015). Some oncogenic plasmids of *Agrobacterium* carry *tzs*, which encodes an IPT that localizes to the bacterial membrane and has been implicated in virulence gene signaling (Aly et al., 2008; Hwang et al., 2013). Recent results showed that genes other than the IPT-encoding *etz* gene of *Pantoea*, are sufficient to cause non-pathogenic strains to elicit gall formation on plants (Lichter et al., 1995; Nissan et al., 2019). Finally, it is particularly noteworthy that cytokinins regulate development of the slime mold, *Dictyostelium discoideum* (Anjard and Loomis, 2008).

The *att* locus represents 10 of the core genes on the virulence plasmids and has been implicated in virulence (Maes et al., 2001). Inconsistent with the reported attenuated phenotype, pFiD188 carrying a non-functional *att* locus is sufficient to transform multiple strains of *Rhodococcus* into being pathogenic (Savory et al., 2017). In addition, *Streptomyces turgidiscabies* strain Car8 can also cause leafy galls on plants (Joshi and Loria, 2007). This strain lacks an *att* locus but has a cluster that includes *fasR, mtr1, mtr2*, and the *fas* genes. The strict conservation of *att* on the three plasmids among plant pathogenic *Rhodococcus* suggests either *att* is maintained because of physical linkage to virulence genes or there is a selective advantage to maintaining the locus. Data are consistent with the possibility that the advantage is unrelated to virulence.

The low number of functions conserved on virulence plasmids of plant pathogenic *Rhodococcus* is consistent with a mechanism of virulence gene cooption (Letek et al., 2010). This model predicts that horizontally acquired genes encode regulators of gene expression. They function to coopt chromosomal genes core to the genus and misregulate their expression to reprogram the recipient genome for virulence. The strongest candidate for cooptive virulence is *fasR* because of its necessity for virulence (Figure 2; Temmerman et al., 2000). Furthermore, the D188 and A21d2 alleles of *fasR* could complement either mutant, and there is potential that FasR recognizes and binds sequences that are conserved among plant associated *Rhodococcus*. Identifying the *fasR-*regulon is important and findings have potential to provide insights into the mechanisms by which virulence plasmids transition *Rhodococcus* to pathogens.

## Data Availability Statement

Biosample IDs for the genome sequences are SAMN02569990 for A21d2 and SAMN0256991 for A25f.

## Author Contributions

ES, AW, AC, and JC conceived and developed the experiments. ES, AW, DS, AC, SF, and EP performed the experiments and analyzed the data. ES, AW, DS, and JC wrote and edited the manuscript.

## Conflict of Interest

The authors declare that the research was conducted in the absence of any commercial or financial relationships that could be construed as a potential conflict of interest.

## Supplementary Material

FIGURE S1 Characterization of the *fasD* mutants. qRT-PCR of the insertion and deletion mutants of *fasD.* The relative-relative expression of *fasA, fasD, fasE*, and *fasF* was determined against expression of the conserved and abundantly expressed Elongation factor Tu (Ef-Tu)-encoding gene and the corresponding gene of D188. The experiment was repeated with similar results.

TABLE S1 Oligonucleotides used in this study.

TABLE S2 Orthogroups containing at least one gene from two or more virulence plasmids.

TABLE S3 TBLASTN results of Leaf str. homologs.
