# Supplementary figures and images for: Phytopathogenic Rhodococcus Have Diverse Plasmids With Few Conserved Virulence Functions
Source: Front Microbiol. 2020 May 25;11:1022. doi: 10.3389/fmicb.2020.01022 (PMC7261884; doi:10.3389/fmicb.2020.01022)

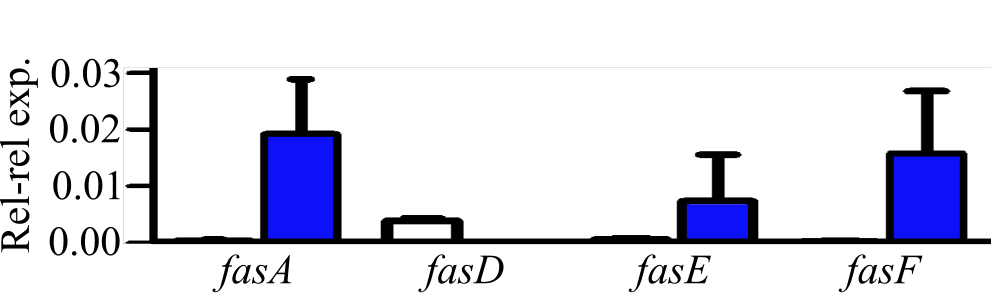

Supplement: FIGURE S1 — Characterization of the fasD mutants. qRT-PCR of the insertion and deletion mutants of fasD. The relative-relative expression of fasA, fasD, fasE, and fasF was determined against expression of the conserved and abundantly expressed Elongation factor Tu (Ef-Tu)-encoding gene and the corresponding gene of D188. The experiment was repeated with similar results. [file Image_1.tiff]
